# CD24 as an innate immune checkpoint in solid tumors: biology, biomarker stratification, and therapeutic translation

**DOI:** 10.3389/fimmu.2026.1798585

**Published:** 2026-03-26

**Authors:** Weiwei Zhou, Xiaomei Huang, Gang Jia, Chuangxin Lu, Wenxue Ma

**Affiliations:** 1 Henan Provincial People’s Clinical Medical School of Zhengzhou University, Zhengzhou, Henan, China; 2 Department of Medical Oncology, Henan Provincial People’s Hospital, People’s Hospital of Zhengzhou University, Zhengzhou, Henan, China; 3 Sanford Stem Cell Institute, Department of Medicine, Moores Cancer Center, University of California San Diego, La Jolla, CA, United States

**Keywords:** cancer immunotherapy, CD24, innate immune checkpoint, myeloid immune suppression, siglec signaling, tumor-associated macrophages

## Abstract

CD24 is a glycosylphosphatidylinositol-anchored surface protein frequently overexpressed in solid tumors and increasingly recognized as an innate immune checkpoint that suppresses macrophage-mediated phagocytosis through engagement of Siglec-10 in humans (Siglec-G in mice). Beyond its associations with tumor aggressiveness and stem-like phenotypes, CD24 functions at a critical interface between tumor-intrinsic plasticity and myeloid-driven immune suppression within the tumor microenvironment (TME). Despite growing therapeutic interest, clinical translation of CD24 targeting has been limited by tumor heterogeneity, redundancy among innate immune checkpoints, safety concerns related to physiological CD24 expression, and the absence of functional biomarker frameworks. In this review, we synthesize recent advances in CD24 biology, biomarker-guided stratification strategies, and emerging CD24-directed therapeutic modalities. We highlight unresolved controversies, define key translational challenges, and propose future directions for integrating CD24 targeting into precision immunotherapy strategies tailored to dominant immune resistance mechanisms in solid tumors.

## Highlights

- CD24 functions as an innate immune checkpoint that suppresses macrophage phagocytosis.
- CD24-Siglec signaling promotes myeloid-driven immune evasion in solid tumors.
- Functional CD24-Siglec activity better predicts therapeutic relevance than CD24 expression alone.
- Effective CD24 targeting requires biomarker-guided and context-dependent strategies.
- Precision-focused trial design is critical for translating CD24-directed immunotherapy.

## 1 Introduction

Solid tumors continue to pose major therapeutic challenges due to their pronounced cellular heterogeneity, adaptive plasticity, and highly immunosuppressive tumor microenvironment (TME) (1, 2). While immune checkpoint blockade targeting adaptive immune pathways such as PD-1/PD-L1 has transformed cancer treatment, durable responses remain limited in many solid malignancies, highlighting the need to identify additional regulatory axes that shape antitumor immunity (3). Increasing attention has therefore shifted toward innate immune checkpoints and soluble mediators that dynamically regulate myeloid cell function, cytokine signaling, and immune-tumor crosstalk within the TME (4).

CD24 has emerged as a notable example of such an innate immune regulatory molecule (5, 6). Initially studied primarily as a tumor-associated marker linked to cancer stemness, metastasis, and poor prognosis, CD24 is now recognized as a functional ligand for Siglec-10 in humans and Siglec-G in murine systems, delivering inhibitory signals that suppress macrophage-mediated phagocytosis (7, 8). This CD24-Siglec axis situates CD24 at a critical convergence point between tumor-intrinsic programs and immune suppression mediated by tumor-associated macrophages (TAMs) (9, 10). A growing body of literature supports a consensus view that CD24 contributes to immune escape in macrophage-rich TME (11, 12); however, important questions remain regarding the relative contribution of CD24 compared with other myeloid checkpoints, its interplay with cytokine-driven macrophage polarization, and the extent to which CD24 signaling influences adaptive immune responses indirectly through myeloid reprogramming (13, 14).

Despite rapid progress, the translational landscape of CD24-targeted therapy remains incompletely defined. Recent studies have introduced diverse therapeutic strategies including bispecific antibodies, supramolecular assemblies, nanotechnology-based signal transducer blockade, oncolytic virus-mediated intratumoral delivery and engineered cellular therapies yet these advances have also exposed key gaps and controversies (15–18). These include variability in CD24 expression across tumor types and disease stages, potential redundancy with parallel immune inhibitory pathways, unresolved safety considerations related to on-target effects in normal tissues, and the absence of standardized biomarker frameworks for patient stratification (13, 19, 20). Addressing these challenges requires a critical synthesis of current evidence that goes beyond descriptive summaries to evaluate mechanistic robustness, therapeutic feasibility, and clinical relevance.

In this review, we aim to integrate emerging insights into CD24 biology with advances in immune engineering approaches and cytokine-mediated immune regulation, critically assessing areas of agreement and disagreement in the literature. By highlighting unresolved questions and defining priorities for future research, we seek to clarify whether and how CD24 can be effectively leveraged as a biomarker and therapeutic target within next-generation, precision-guided immunotherapy strategies for solid tumors.

## 2 Biological functions of CD24 in solid tumors: mechanistic insights and unresolved questions

Evidence supporting the biological and therapeutic relevance of CD24 spans multiple experimental systems, including mechanistic *in vitro* studies, preclinical animal models, and limited analyses of human tumor samples (13, 21). Throughout this section, we distinguish these levels of evidence to clarify the current maturity of the CD24 field.

### 2.1 Molecular features and expression landscape of CD24

CD24 is a small, heavily glycosylated glycosylphosphatidylinositol (GPI)-anchored surface protein expressed across a range of epithelial tissues, immune cell subsets, and stem-like cellular compartments (22–24). In solid tumors, CD24 is frequently upregulated and exhibits marked heterogeneity both across cancer types and within individual tumors (15, 25, 26). Multiple studies report that high CD24 expression is associated with aggressive clinicopathological features, including enhanced invasion, metastatic propensity, and poor patient outcomes in malignancies such as breast, ovarian, pancreatic, hepatocellular, and colorectal cancers (6, 7, 18, 27–30).

Despite this consensus, the functional interpretation of CD24 expression remains complex (31). CD24 lacks an intracellular signaling domain, suggesting that its biological effects are mediated through extracellular interactions, membrane organization, and cooperation with other surface receptors (32). Moreover, CD24 glycosylation patterns, which critically influence ligand binding are dynamic and context dependent yet remain insufficiently characterized in most tumor settings (21, 33). This variability represents an underappreciated source of biological heterogeneity and may partially explain inconsistent correlations between CD24 expression levels and therapeutic response across studies.

### 2.2 CD24 as an innate immune checkpoint: interaction with Siglec-10/G

CD24 engages inhibitory Siglec receptors Siglec-10 in humans and Siglec-G in murine systems on myeloid cells, triggering intracellular signaling events that suppress phagocytic activation (9). Ligand binding induces recruitment of the phosphatases SHP-1 and SHP-2, which attenuate activating signals downstream of Fc receptors and pattern recognition receptors (34, 35). Through this pathway, CD24 signaling interferes with cytoskeletal rearrangement and engulfment programs required for effective macrophage-mediated clearance (36, 37).

Mechanistically, the CD24-Siglec axis exhibits specificity for damage-associated molecular pattern (DAMP)-driven activation rather than broadly suppressing basal phagocytic capacity (9, 38). In contrast to the CD47-SIRPα pathway (39), which inhibits phagocytosis across diverse contexts (40, 41), CD24 preferentially dampens macrophage responses to inflammatory danger signals, allowing sustained tolerance to stressed or dying cells in inflammatory environments (42, 43). The relative contribution of CD24-mediated signaling versus other inhibitory myeloid checkpoints varies across tumor types, raising questions regarding pathway dominance and the extent to which SHP-dependent signaling redundancy may necessitate combinatorial targeting strategies (5, 13).

Current evidence supporting CD24 as an innate immune checkpoint derives primarily from mechanistic and preclinical studies (21). *In vitro* experiments demonstrate that CD24 engagement with Siglec-10 suppresses macrophage phagocytosis through recruitment of SHP-1 and SHP-2 phosphatases (5). Mouse models further support a role for the CD24-Siglec-G axis in regulating macrophage-mediated immune surveillance (44, 45). In contrast, clinical evidence remains limited to correlative observations in human tumor samples (13, 19, 46), and large-scale clinical validation comparable to the CD47-SIRPα pathway has not yet been established.

While both the CD24-Siglec and CD47-SIRPα pathways function as macrophage phagocytosis checkpoints (5, 13, 39), they differ substantially in expression patterns, translational maturity, and safety considerations (31, 36), with CD47-directed therapeutics already entering multiple clinical trials whereas CD24-targeted strategies remain largely at the preclinical stage. CD47 is broadly expressed across tumor types and has advanced into multiple clinical trials (47, 48), whereas CD24-targeted therapeutic strategies remain largely at the preclinical or early translational stage (15, 49). In addition, CD47 blockade is associated with hematologic toxicities due to erythrocyte expression (50, 51), while the safety profile of systemic CD24 inhibition remains less well defined but raises concerns due to its expression in normal epithelial and immune cells (13, 21).

### 2.3 Integration of CD24 signaling with cytokine networks and macrophage polarization

Beyond direct checkpoint signaling, CD24 operates within a broader immunoregulatory network shaped by cytokines, soluble mediators, and metabolic cues in the TME (13, 52). TAMs frequently express high levels of Siglec-10, are themselves molded by cytokines such as IL-10, TGF-β, CSF-1, and IL-6, all of which can reinforce immunosuppressive phenotypes (53, 54). CD24-mediated inhibitory signaling may therefore function as a downstream amplifier of cytokine-driven macrophage polarization rather than as an isolated suppressive mechanism (55).

A critical gap in current knowledge is how CD24-Siglec signaling intersects with dynamic cytokine gradients and whether CD24 blockade reshapes macrophage function indirectly by altering cytokine production, antigen presentation capacity, or metabolic programming (5, 56). While emerging studies suggest that CD24 inhibition may promote a more pro-inflammatory macrophage phenotype with enhanced phagocytic activity, the durability and spatial specificity of these effects remain poorly defined (57, 58). Dissecting these interactions will be essential for understanding how CD24 targeting influences the broader immune ecosystem of solid tumors.

### 2.4 CD24, tumor plasticity, and cancer stem-like states

CD24 is frequently co-expressed with markers associated with tumor plasticity and cancer stem-like phenotypes, including CD44 and CD133 (59, 60). This association has led to the prevailing view that CD24 contributes to therapeutic resistance, tumor relapse, and metastatic dissemination. Experimental evidence supports a role for CD24 in enhancing cell adhesion, migration, and invasion through interactions with selectins, integrins, and extracellular matrix components, as well as through activation of pathways such as STAT3, FAK, and PI3K-AKT (21, 61).

However, the relationship between CD24 expression and stemness remains context dependent and, in some cases, contradictory (62). In certain tumor models, CD24 marks highly plastic, therapy-resistant subpopulations, whereas in others it is expressed more broadly across differentiated tumor cells (7, 19, 63). These discrepancies highlight the need to move beyond binary classifications of CD24 positivity and instead consider quantitative expression levels, co-marker profiles, and spatial localization within the tumor architecture.

Emerging evidence also suggests that CD24 may contribute to resistance to targeted therapies (7, 16). For example, recent studies in EGFR-mutated lung cancer indicate that CD24 expression may reduce sensitivity to EGFR tyrosine kinase inhibitors by promoting immune evasion and tumor cell plasticity (15). These findings suggest that CD24 targeting may enhance the efficacy of targeted therapies in addition to its role in immunotherapy strategies.

### 2.5 Key controversies and unresolved gaps in CD24 biology

Despite substantial progress, several fundamental questions remain unanswered. First, it is unclear whether CD24 primarily functions as a driver of tumor aggressiveness or as a context-dependent facilitator that amplifies existing oncogenic and immunosuppressive programs (64, 65). Second, the extent to which CD24 signaling influences adaptive immunity either indirectly through myeloid cells or directly via interactions with T-cell-expressed Siglecs remains an area of active investigation (21, 56). Third, the lack of standardized assays to assess functional CD24-Siglec engagement, rather than static CD24 expression alone, limits translational interpretation of existing studies (24).

Addressing these gaps will require integrative approaches combining spatial transcriptomics, proteomics, and functional immune profiling to map CD24 activity within the evolving TME (64). Such efforts are critical for determining when CD24 represents a dominant immunosuppressive axis and when it acts as one component of a redundant checkpoint network (13, 19).

**Figure 1:**
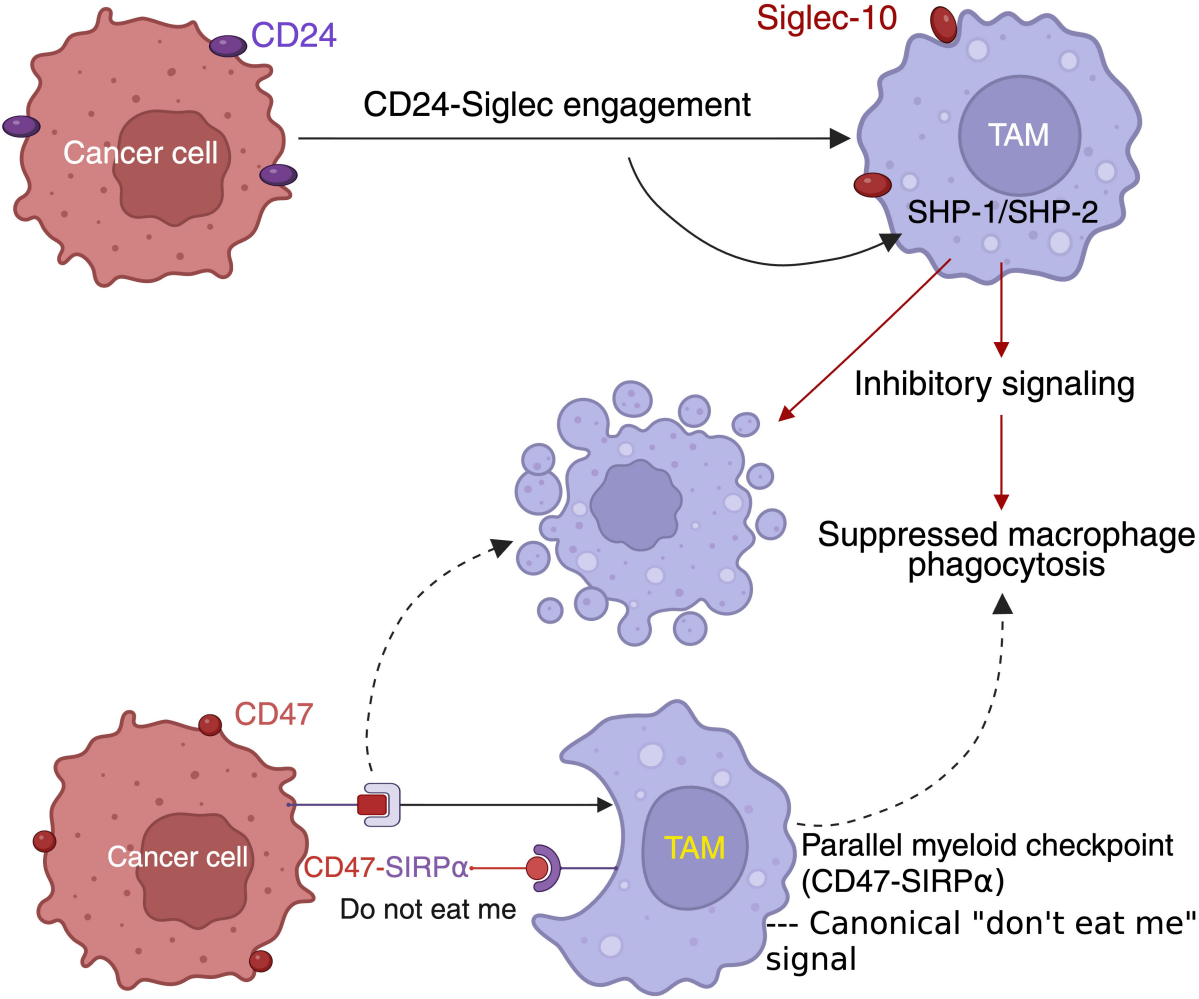
CD24 functions as an innate immune checkpoint suppressing macrophage phagocytosis in solid tumors. Schematic illustrating CD24-mediated innate immune suppression within the TME. CD24 expressed on tumor cells engages Siglec-10 (human) on TAMs, triggering ITIM-dependent recruitment of inhibitory phosphatases SHP-1 and SHP-2 and downstream inhibitory signaling that suppresses macrophage phagocytosis. This CD24-Siglec axis converges functionally with the canonical myeloid checkpoint CD47-SIRPα, which similarly delivers a “don’t eat me” signal through shared antiphagocytic signaling pathways, thereby promoting immune evasion in macrophage-rich solid tumors.

Together, these mechanistic insights position CD24 as a context-dependent regulator of tumor-immune interactions, providing the biological foundation for emerging therapeutic strategies that aim to disrupt CD24-mediated immune suppression in solid tumors (21, 66). These mechanistic interactions underlying CD24-mediated innate immune suppression are schematically summarized in **Figure 1**.

## 3 CD24 as a biomarker in solid tumors: prognostic value, predictive potential, and translational challenges

### 3.1 Prognostic significance of CD24 expression in solid tumors

CD24 has been extensively investigated as a prognostic biomarker across a broad spectrum of solid tumors, including breast, ovarian, pancreatic, colorectal, hepatocellular, and renal malignancies (6, 7, 24, 67). A general consensus has emerged that elevated CD24 expression is associated with adverse clinical outcomes, such as increased tumor aggressiveness, metastatic potential, and reduced overall survival (68, 69). These associations are consistent with the biological roles of CD24 in tumor plasticity and immune evasion, reinforcing its relevance as a marker of high-risk disease (19, 70, 71).

However, the prognostic utility of CD24 is not uniform across tumor types or clinical contexts (6). Variability in detection methods, scoring thresholds, and tissue sampling strategies has led to inconsistent conclusions in some studies (72). Moreover, CD24 expression often overlaps with other high-risk features, raising questions about its independence as a prognostic factor (73, 74). These limitations underscore the need for standardized assays and multivariate analyses to determine whether CD24 adds prognostic value beyond established clinical and molecular parameters.

To synthesize the reported patterns of CD24 expression and their clinical associations across solid tumors, key findings from representative studies are summarized in **Table 1**.

**Table 1:** CD24 expression and clinical relevance across solid tumors.

Tumor type	CD24 expression pattern	Clinical associations	Key notes and limitations	References
Breast cancer	Frequently overexpressed, particularly in aggressive subtypes	Poor prognosis, increased metastasis, therapy resistance	Expression varies across subtypes; functional role linked to immune evasion and stem-like states	(27, 75)
Triple-negative breast cancer (TNBC)	High CD24 expression in subsets	Tumor aggressiveness, immune suppression	Not uniformly expressed; heterogeneity complicates patient stratification	(76, 77)
Ovarian cancer	Elevated CD24 in poorly differentiated tumors	Advanced stage, reduced survival	Prognostic value varies among cohorts	(78, 79)
Pancreatic ductal adenocarcinoma	High and heterogeneous CD24 expression	Invasion, metastasis, poor outcome	Strong stromal influence; difficult to isolate tumor-intrinsic effects	(80, 81)
Hepatocellular carcinoma	Upregulated CD24 in tumor tissue	Tumor progression, unfavorable prognosis	Often co-expressed with stemness markers	(28, 82)
Colorectal cancer	Increased CD24 in advanced disease	Lymph node metastasis, reduced survival	Expression differs between primary and metastatic lesions	(29, 83)
Gastric cancer	CD24 overexpression reported	Advanced stage, poor differentiation	Limited large-scale validation studies	(22, 23)
Lung cancer (NSCLC)	Variable CD24 expression	Association with metastasis and prognosis reported	Conflicting results across studies	(15, 84)
Renal cell carcinoma	Elevated CD24 in subsets	Poor prognosis	Mechanistic role remains unclear	(85, 86)
Head and neck squamous cell carcinoma	CD24 expression in tumor cells	Aggressive behavior, nodal involvement	Data remain limited	(87, 88)

### 3.2 Beyond expression: functional and spatial biomarkers of the CD24-Siglec axis

While most clinical studies have relied on static measurements of CD24 expression, emerging evidence suggests that functional engagement of the CD24-Siglec axis, rather than CD24 abundance alone, may be more relevant for therapeutic decision-making (5, 6). From a biomarker perspective, this distinction is critical. CD24-mediated immune suppression depends on the presence of Siglec-10/G-expressing myeloid cells and their spatial proximity to CD24-positive tumor cells within the TME (15, 56).

This realization has shifted attention toward composite biomarker strategies that integrate CD24 expression with myeloid cell profiling (74, 89). Parameters such as tumor-associated macrophage density, Siglec-10 expression levels, macrophage polarization state, and spatial organization relative to tumor nests may better capture the functional status of the CD24 checkpoint (13, 90). However, these multidimensional biomarkers are rarely incorporated into current clinical workflows, representing a major translational gap.

Several experimental approaches may facilitate detection of functional CD24-Siglec signaling activity in clinical samples (5, 21). Multiplex immunohistochemistry or spatial proteomics can quantify tumor CD24 expression and assess spatial proximity to Siglec-10-positive macrophages (5, 15). Single-cell RNA sequencing and spatial transcriptomics may further characterize myeloid cell states and pathway activation signatures within the tumor microenvironment (91, 92). Functional assays measuring macrophage phagocytic activity in CD24-high tumor contexts may also provide complementary evidence of pathway engagement. Integration of these approaches could enable clinically actionable biomarker strategies (93).

### 3.3 Predictive value of CD24 for immunotherapy and combination strategies

The predictive role of CD24 in guiding therapeutic response remains an area of active investigation (6, 7). Preclinical studies increasingly support the notion that tumors enriched for CD24, and Siglec-10-positive macrophages may be particularly susceptible to CD24-targeted interventions, especially when combined with other immunomodulatory strategies (5, 94). Conversely, tumors dominated by adaptive immune resistance mechanisms or low macrophage infiltration may derive limited benefit from CD24 blockade alone (13, 95).

Importantly, CD24 expression has not yet been systematically evaluated as a predictive biomarker for response to established immune checkpoint inhibitors, such as PD-1 or PD-L1 blockade, in large clinical cohorts (13, 96). This represents a critical knowledge gap, as CD24-driven myeloid suppression may contribute to primary or acquired resistance to T cell-directed immunotherapies (19, 94, 97). Prospective studies integrating CD24-axis biomarkers into immunotherapy trials are therefore needed to clarify its predictive utility (18).

### 3.4 CD24 in the context of tumor heterogeneity and temporal dynamics

Tumor heterogeneity and temporal evolution pose significant challenges to biomarker implementation (98). CD24 expression can vary not only between patients but also within different regions of the same tumor and across disease stages or treatment courses (13, 99). This spatial and temporal variability limits the reliability of single-biopsy assessments and highlights the importance of longitudinal and multi-region sampling strategies.

Advanced technologies such as spatial transcriptomics, multiplex immunohistochemistry, and single-cell profiling offer powerful tools to address these challenges (100). By capturing dynamic changes in CD24 expression and myeloid cell composition over time, these approaches may enable more accurate patient stratification and real-time monitoring of therapeutic responses (101). Nonetheless, translating these technologies into routine clinical practice will require simplification, standardization, and validation in prospective trials.

### 3.5 Toward a biomarker-guided framework for CD24-targeted therapy

Collectively, current evidence supports CD24 as a biologically meaningful but context-dependent biomarker in solid tumors. Its greatest potential lies not as a standalone marker but as part of an integrated biomarker framework that reflects tumor-intrinsic features, immune cell composition, and spatial organization within the TME (6, 21, 24). Such a framework could guide patient selection for CD24-targeted therapies, inform rational combination strategies, and improve risk stratification.

Future efforts should prioritize the development of standardized assays to assess CD24-Siglec pathway activity, the incorporation of myeloid and cytokine-related biomarkers, and the validation of these markers in clinically annotated cohorts (102). By aligning biomarker development with the underlying biology of CD24-mediated immune suppression, the field can move toward more precise and effective application of CD24-targeted immunotherapy (21, 66).

**Figure 2:**
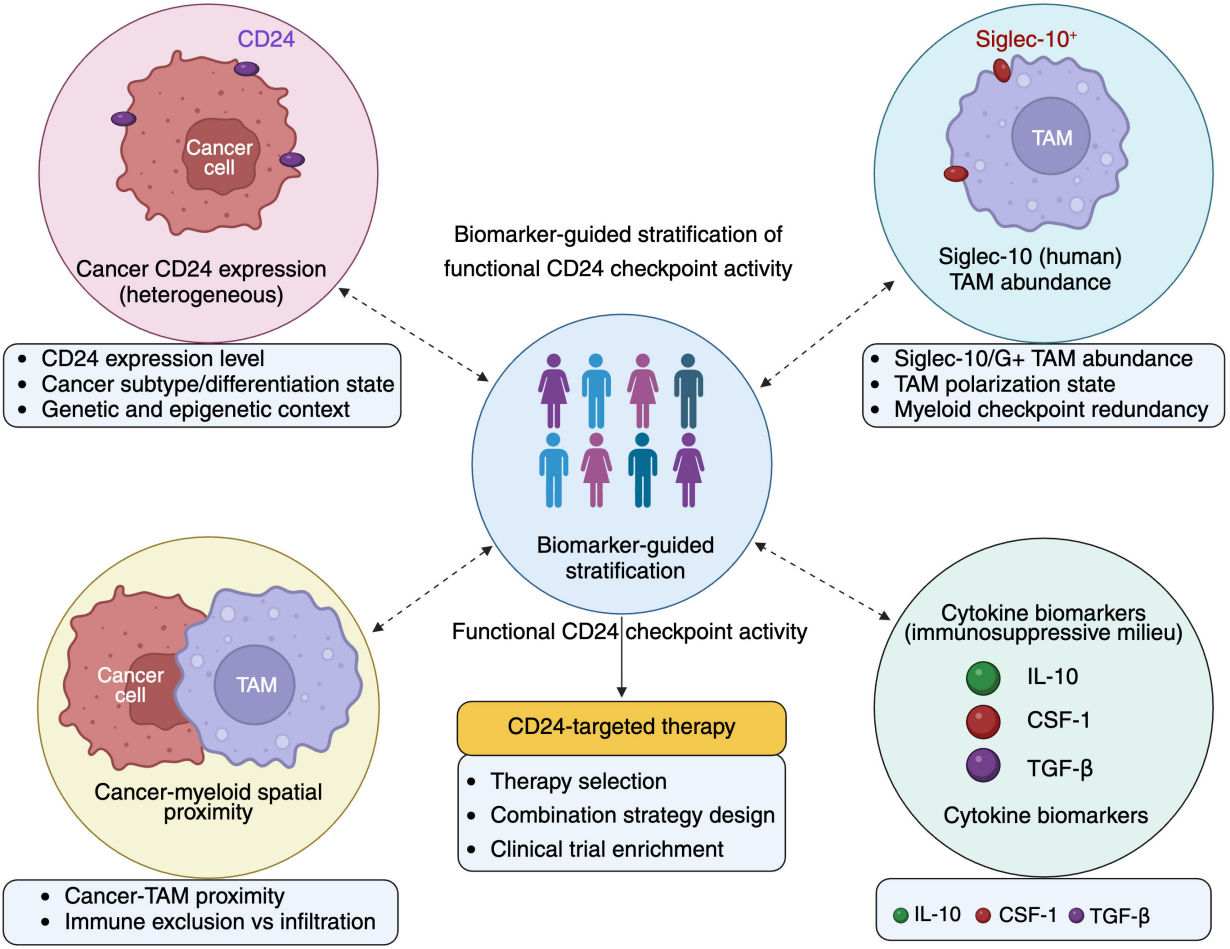
Biomarker framework for functional stratification of CD24-dependent immune suppression in solid tumors. Conceptual framework illustrating biomarker-guided stratification of tumors based on functional CD24-mediated innate immune checkpoint activity. Effective CD24 checkpoint signaling is determined by the integration of multiple tumor- and microenvironmental parameters, including heterogeneous tumor CD24 expression, abundance and functional state of Siglec-10/G-positive TAMs, spatial proximity between tumor cells and myeloid populations, and the surrounding immunosuppressive cytokine milieu (e.g., IL-10, CSF-1, TGF-β). Integrative assessment of these features enables identification of tumors with dominant CD24-dependent immune suppression and supports rational patient selection, combination strategy design, and clinical deployment of CD24-targeted therapies.

These biomarker considerations provide a critical foundation for evaluating emerging CD24-targeted therapeutic strategies and for designing rational, biomarker-informed clinical interventions (66, 103). An integrated, biomarker-guided framework for stratifying CD24-dependent immune suppression is summarized in **Figure 2**.

## 4 Therapeutic targeting of CD24: modality-biomarker alignment and translational logic

**Figure 3:**
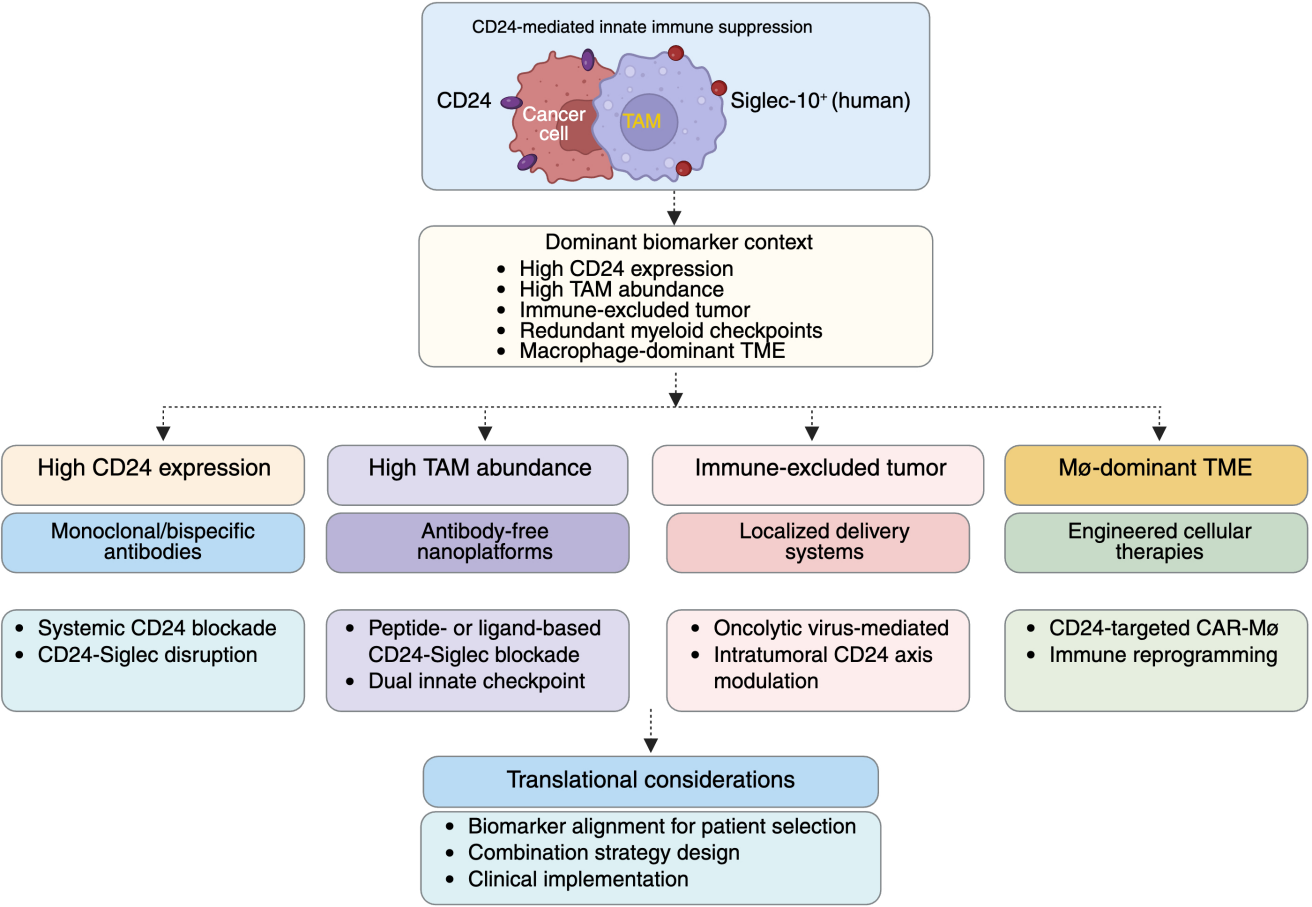
Therapeutic strategies targeting the CD24 axis aligned with biomarker context in solid tumors. Schematic illustrating biomarker-informed alignment of emerging therapeutic modalities targeting CD24-mediated innate immune suppression. Distinct CD24-targeted strategies are mapped to dominant biomarker contexts, including high tumor CD24 expression, high Siglec-10/G-positive tumor-associated macrophage abundance, immune-excluded tumor phenotypes, and macrophage-dominant TMEs. Therapeutic approaches encompass monoclonal and bispecific antibodies, antibody-free nanotechnology-based platforms, localized delivery systems such as oncolytic virus-mediated intratumoral modulation and engineered cellular therapies. Integration of biomarker context with therapeutic modality selection provides a translational framework to guide patient selection, rational combination strategy design, and clinical implementation of CD24-targeted immunotherapies.

Guided by emerging biomarker insights, a growing diversity of CD24-targeted therapeutic strategies has emerged, reflecting both the biological complexity of the CD24 axis and the need for context-specific intervention (66, 104). Importantly, the effectiveness of each modality is likely to depend on distinct biomarker features, underscoring the necessity of aligning therapeutic design with biological and immunological context (49). **Figure 3** summarizes emerging CD24-targeted therapeutic modalities and illustrates how distinct biomarker contexts may guide modality selection and translational deployment in solid tumors.

### 4.1 Monoclonal antibodies and bispecific strategies targeting CD24

Antibody-based approaches targeting CD24 represent the most direct strategy to disrupt CD24-mediated innate immune suppression. Preclinical studies demonstrate that CD24-targeting antibodies can enhance macrophage-mediated phagocytosis and suppress tumor growth, particularly in tumors enriched for macrophages (13, 15, 105). However, because CD24 functions within a broader network of redundant myeloid checkpoints, blockade of CD24 alone may be insufficient to fully restore phagocytic activity in many tumor contexts (13, 14, 94). This limitation has motivated the development of bispecific strategies, most notably dual CD24/CD47 targeting approaches, designed to simultaneously relieve multiple inhibitory signals governing macrophage function (26, 106). Such strategies are most rational in tumors characterized by high CD24 expression, abundant Siglec-10-positive macrophages, and concurrent activation of the CD47-SIRPα axis. Despite their promise, antibody-based and bispecific approaches face important translational challenges, including systemic toxicity, on-target effects in normal tissues expressing CD24, and the need to achieve selective engagement within the TME (26, 107, 108).

### 4.2 Antibody-free and nanotechnology-enabled CD24-Siglec blockade

Antibody-free approaches have recently emerged as alternative strategies to disrupt CD24-Siglec interactions, with nanotechnology-enabled platforms offering translational flexibility (5, 38, 49, 109). Signal-transducer peptide-anchored nanoparticles capable of competitively blocking CD24-Siglec-10/G engagement have been shown to activate macrophages while allowing modular design features, including the co-delivery of agents targeting complementary myeloid checkpoints such as CD47-SIRPα (20, 64). These platforms are best suited for tumors in which functional CD24-Siglec signaling and macrophage-dominant immune suppression can be demonstrated, rather than tumors driven primarily by adaptive immune resistance. Compared with systemic antibody administration, nanotechnology-based approaches may reduce reliance on prolonged antibody exposure and enable integrated combination strategies within a single delivery system (110, 111). Nonetheless, key translational uncertainties remain, particularly with respect to biodistribution, targeting specificity, and the long-term immunological consequences of sustained innate immune modulation (112, 113).

### 4.3 Localized delivery platforms: oncolytic viruses and intratumoral “factory” strategies

Localized delivery platforms, particularly oncolytic virus-based systems engineered to release CD24- or Siglec-targeting fusion proteins within the tumor, represent an approach to spatially restrict immune modulation and limit systemic exposure (114, 115). By confining checkpoint disruption to the tumor site, these strategies aim to reprogram TAMs while simultaneously leveraging virus-induced inflammation and antigen release to amplify innate immune activation (42, 116). Such platforms are especially well suited for accessible tumors characterized by high CD24 expression and dense myeloid infiltration, where local macrophage re-education may meaningfully alter the tumor immune landscape (54, 117, 118). Despite their conceptual appeal, challenges related to scalability, patient selection, and the durability of macrophage reprogramming remain unresolved and will be critical determinants of clinical feasibility (119).

### 4.4 CD24-targeted cellular therapies

CD24-targeted cellular therapies, including CD24-directed CAR-T cells, extend CD24 intervention beyond myeloid checkpoint modulation to direct tumor cell elimination (120). Preclinical studies suggest that these approaches may not only mediate cytotoxic clearance of CD24-expressing tumor cells but also promote secondary immune activation through antigen spreading and remodeling of the TME (121). These strategies are most applicable to tumors with relatively homogeneous or dominant CD24 expression and limited antigen heterogeneity, where target loss is less likely to undermine efficacy (120, 122). However, significant risks remain, including antigen escape, off-tumor toxicity due to CD24 expression in normal tissues, and impaired persistence or function within highly immunosuppressive TMEs (13, 123). Future iterations are likely to rely on logic-gated or combinatorial CAR designs that integrate myeloid checkpoint modulation to enhance both specificity and durability (124, 125).

### 4.5 Rational combination strategies informed by CD24 biology

Given the redundancy and plasticity of immune suppressive pathways in solid tumors, CD24-targeted therapies are unlikely to achieve durable efficacy as monotherapies in most clinical contexts (13, 19). Rational combination strategies should therefore be guided by biomarker evidence indicating dominant myeloid-driven immune suppression (5, 126). In tumors with high CD24 expression and macrophage-rich microenvironments, CD24-centered combinations may be sufficient to restore innate immune clearance, whereas tumors co-expressing CD24 and CD47 may require dual myeloid checkpoint targeting to overcome parallel inhibitory signals (26, 50). In settings characterized by CD24-driven macrophage suppression and poor T-cell infiltration, macrophage priming through CD24 blockade may be necessary before effective engagement of adaptive immune therapies (40, 127). Collectively, these considerations reinforce the view of CD24 as a context-dependent immunoregulatory node, highlighting the need for biomarker-aligned therapeutic design, safety-forward engineering, and spatially informed patient selection to enable successful clinical translation (15, 66, 128).

Despite the expanding therapeutic toolkit for targeting CD24, significant biological, technical, and clinical constraints continue to limit effective translation, necessitating a focused examination of the challenges that define the path forward (123, 129).

At present, most CD24-targeted therapeutic strategies remain in preclinical development (49, 102). Compared with CD47-targeted therapies, which have progressed into multiple early-phase clinical trials, CD24-directed agents are still largely undergoing experimental validation (15, 130). Nevertheless, increasing interest from academic groups and biotechnology companies suggests that early-phase clinical evaluation may emerge in the near future (131–134). Continued translational research will be necessary to establish safety, dosing strategies, and biomarker-guided patient selection.

Recent CD24-targeted therapeutic strategies spanning multiple modalities and experimental systems, are summarized in **Table 2**, which highlights that most approaches remain at the preclinical or early translational stage, with limited clinical development reported to date.

**Table 2:** Current preclinical and emerging therapeutic strategies targeting CD24 in solid tumors.

Therapeutic modality	Targeted axis	Delivery/platform	Experimental context	Development stage	Key findings	Translational considerations	References
Monoclonal antibodies	CD24	Systemic antibody administration	*In vitro* and preclinical tumor models	Preclinical (*in vitro* + *in vivo*)	Enhanced macrophage phagocytosis; tumor growth inhibition	Risk of on-target off-tumor effects; checkpoint redundancy	(13, 41)
Bispecific antibodies	CD24 + CD47	Dual-target antibody constructs	Preclinical solid tumor models	Preclinical	Improved phagocytosis via dual checkpoint disruption	Potential hematologic toxicity; requires biomarker selection	(26, 106)
Signal-transducer nanoparticles	CD24-Siglec-10/G	Antibody-free nanomedicine	Breast cancer models	Preclinical (*in vivo*)	Macrophage activation and tumor suppression	Biodistribution and long-term safety remain unclear	(64, 135)
Supramolecular peptide-antibody assemblies	CD24 + CD47	*In situ* self-assembly platforms	Preclinical solid tumor models	Preclinical (*in vivo*)	Synergistic blockade of innate checkpoints	Manufacturing complexity; regulatory considerations	(58, 136)
Oncolytic virus-mediated delivery	Siglec-10/G and/or CD24	Intratumoral viral “factory” systems	Murine and humanized tumor models	Preclinical (*in vivo*)	Local macrophage reprogramming; tumor regression	Limited to accessible tumors; scalability challenges	(42, 65)
CD24-targeted CAR-T cells	CD24	Engineered cellular therapy	Preclinical solid tumor models	Preclinical (early translational research)	Direct tumor killing and immune activation	Antigen heterogeneity; off-tumor toxicity risk	(17, 120)
RNA interference approaches	CD24	siRNA or vector-based delivery	Preclinical tumor models	Preclinical (*in vivo*)	Reduced CD24 expression; increased therapy sensitivity	Delivery efficiency and off-target effects	(137, 138)
Combination with immune checkpoint inhibitors	CD24 + PD-1/PD-L1	Systemic combination therapy	Preclinical immunotherapy models	Preclinical (*in vivo*)	Enhanced antitumor immunity in select contexts	Requires careful patient stratification	(34, 139)
Combination with chemo or radiotherapy	CD24-centered combinations	Multimodal treatment regimens	Preclinical solid tumor models	Preclinical (*in vivo*)	Increased immune-mediated tumor control	Timing and toxicity optimization needed	(140, 141)

## 5 Translational challenges, safety considerations, and resistance mechanisms

Despite rapid progress in CD24-targeted therapeutic development, multiple biological and translational challenges must be addressed before these strategies can be successfully integrated into clinical practice. These challenges reflect not only the intrinsic complexity of CD24 biology, but also broader limitations associated with targeting innate immune checkpoints in solid tumors (5, 7).

### 5.1 On-target off-tumor effects and safety considerations

A fundamental concern in CD24-targeted therapy is the lack of tumor exclusivity (66). CD24 is expressed on various normal epithelial and immune cell populations, raising the risk of on-target off-tumor toxicity (15, 24). Unlike adaptive immune checkpoints, whose expression is often induced in pathological contexts, CD24 plays physiological roles in immune regulation and tissue homeostasis (6, 13). This complicates systemic targeting approaches, particularly antibody-based strategies.

Emerging delivery platforms such as intratumoral “factory” systems, nanoparticle-based formulations, and logic-gated cellular therapies, partially address this issue by restricting CD24 modulation to the TME (5, 142). However, the long-term consequences of sustained CD24-Siglec disruption on systemic immune tolerance, inflammatory balance, and tissue repair remain poorly understood (9, 56). Rigorous safety profiling and careful dose optimization will therefore be essential in early-phase clinical development.

Potential toxicities associated with systemic CD24 blockade may include disruption of immune tolerance mechanisms, aberrant inflammatory responses, and impaired tissue repair processes (102, 143). CD24 has been implicated in the regulation of damage-associated molecular pattern (DAMP) signaling and the maintenance of immune homeostasis; therefore, sustained inhibition of CD24 signaling may increase susceptibility to autoimmune or inflammatory complications (6, 9, 38).

### 5.2 Tumor heterogeneity and antigen escape

Intratumoral heterogeneity poses a major barrier to durable responses (144, 145). CD24 expression varies spatially within tumors and temporally during disease progression and treatment (146). Therapeutic pressure may select for CD24-low or CD24-negative subclones, leading to immune escape and disease relapse (63, 76). This risk is particularly relevant for CD24-targeted cellular therapies and monotherapy approaches.

Moreover, CD24 functions within a network of redundant immune inhibitory pathways (7, 13). Tumors may compensate for CD24 blockade by upregulating alternative myeloid checkpoints, such as CD47-SIRPα or inhibitory LILRB family members (14, 106). These adaptive resistance mechanisms underscore the importance of combination strategies and longitudinal monitoring to detect pathway reprogramming during treatment.

### 5.3 Context dependency of myeloid checkpoint targeting

Not all tumors are equally dependent on myeloid-driven immune suppression (147). Tumors dominated by adaptive immune exclusion, stromal barriers, or metabolic constraints may derive limited benefit from CD24 targeting alone (102). In such contexts, CD24-directed therapies may fail to produce meaningful immune reactivation despite effective target engagement (21, 24).

This context dependency highlights the need for biomarker-informed patient selection. Without appropriate stratification, negative trial outcomes may reflect suboptimal cohort selection rather than true lack of therapeutic potential (148). The challenge lies in defining clinically feasible assays that capture functional myeloid suppression rather than static marker expression (19).

### 5.4 Incomplete understanding of systemic immune consequences

Although CD24-Siglec signaling is primarily studied in macrophages, Siglec family members are expressed on multiple immune cell subsets, including dendritic cells and certain T-cell populations (5, 149). Disrupting this pathway may therefore have broader immunological consequences beyond phagocytosis, potentially influencing antigen presentation, cytokine production, and immune homeostasis (34, 150).

At present, the systemic immunological effects of sustained CD24 blockade particularly in combination regimens remain insufficiently characterized (6, 19, 52). Preclinical models often fail to fully recapitulate human immune complexity, emphasizing the need for translational studies incorporating humanized systems and immune monitoring in early clinical trials (151, 152).

### 5.5 Regulatory, manufacturing, and clinical trial design challenges

From a translational standpoint, several practical challenges must also be considered. Complex biologics, nanomedicine platforms, and engineered cellular therapies pose manufacturing, scalability, and regulatory hurdles (153, 154). Standardizing production while preserving functional activity and safety will be critical for clinical advancement. Clinical trial design presents an additional obstacle. Traditional response endpoints may not adequately capture the immunomodulatory effects of CD24-targeted therapies, particularly those acting on myeloid cells (19, 24). Incorporating immune-based endpoints, spatial biomarker analyses, and adaptive trial designs may be necessary to accurately assess therapeutic benefit (155, 156). Overcoming these limitations will require not only technological innovation but also a forward-looking research agenda that integrates systems-level immune analysis, biomarker development, and precision trial design (157).

**Figure 4:**
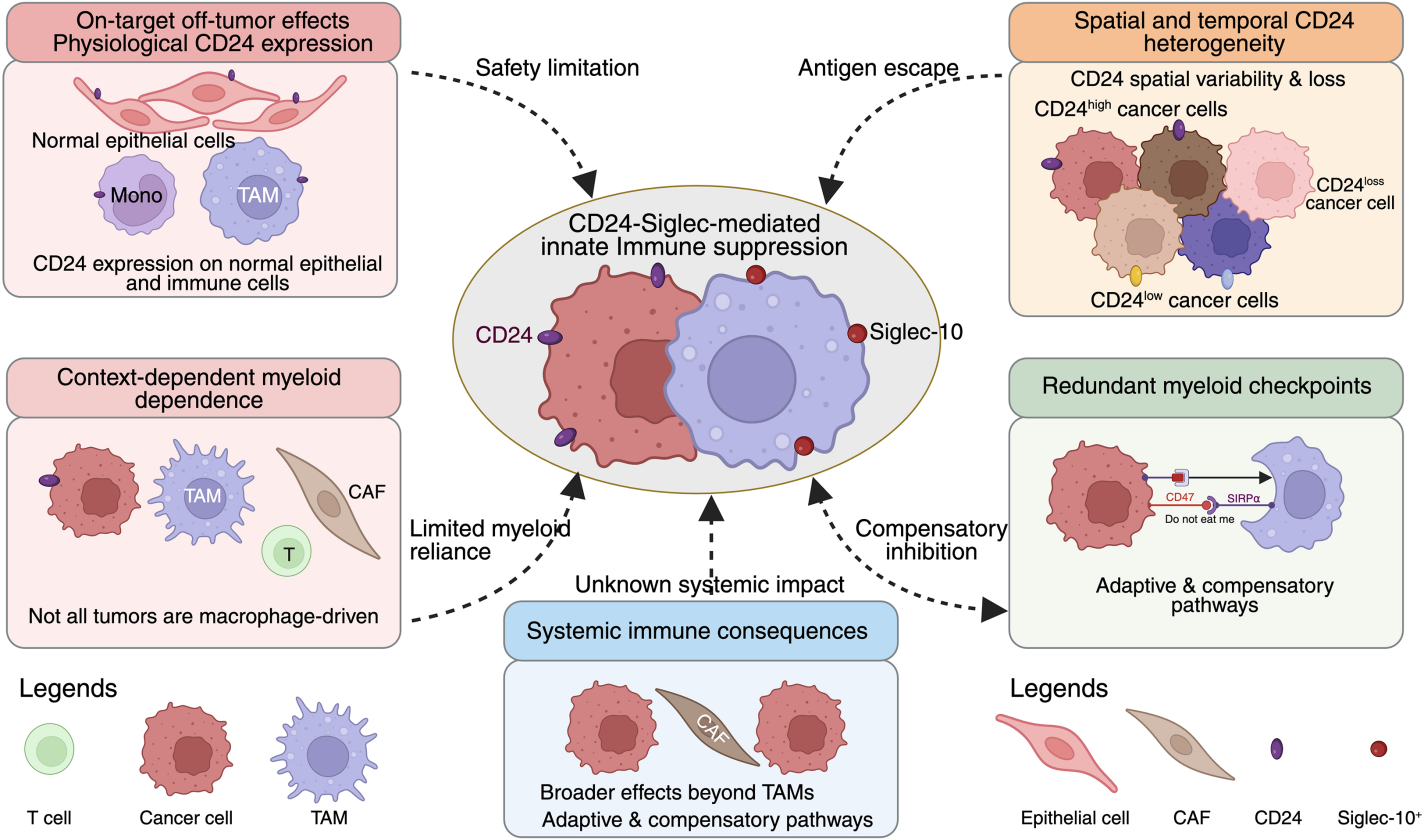
Translational challenges and resistance mechanisms limiting CD24-targeted immunotherapy. Schematic illustrating the major biological and translational constraints that limit the efficacy and durability of CD24-directed immunotherapy in solid tumors. Key barriers include on-target off-tumor effects arising from physiological CD24 expression on normal epithelial and immune cells, spatial and temporal heterogeneity of tumor CD24 expression leading to antigen escape, and compensatory activation of redundant innate myeloid checkpoints such as the CD47-SIRPα axis. Therapeutic efficacy is further shaped by context-dependent reliance on macrophage-mediated immune suppression and by incomplete understanding of systemic immune consequences following CD24-Siglec pathway disruption. Together, these convergent challenges highlight the need for biomarker-guided patient stratification, rational combination strategies, and safety-focused translational development to enable effective clinical deployment of CD24-targeted therapies.

These translational and clinical considerations are summarized in **Figure 4**, which integrates the major biological, safety, and resistance-related barriers that currently constrain the efficacy and durability of CD24-targeted immunotherapy in solid tumors.

## 6 Future directions and research priorities

The evolving understanding of CD24 as an innate immune checkpoint highlights both the promise and complexity of targeting this pathway in solid tumors (34, 102). Moving forward, progress in this field will depend on integrating mechanistic biology with systems-level analysis, biomarker refinement, and precision-driven clinical trial design (158).

### 6.1 Defining functional CD24-Siglec signaling states in the TME

A key priority for future research is to move beyond static measurements of CD24 expression toward defining functional CD24-Siglec signaling states within tumors (5). This will require experimental frameworks capable of capturing real-time interactions between CD24-positive tumor cells and Siglec-10/G-expressing myeloid cells, as well as downstream signaling consequences (56). Functional assays that assess macrophage phagocytic capacity, inhibitory phosphatase recruitment, and cytokine output *in situ* will be essential for distinguishing tumors that are truly dependent on CD24-mediated immune suppression from those in which CD24 plays a secondary role (7, 13).

### 6.2 Integrating cytokine signaling and soluble mediators into CD24 biology

CD24 does not operate in isolation but within a cytokine- and chemokine-rich microenvironment that dynamically shapes immune cell behavior (19, 46). Future studies should systematically examine how CD24-Siglec signaling interfaces with cytokine networks that regulate macrophage polarization, immune cell recruitment, and inflammatory tone, including pathways driven by IL-10, TGF-β, CSF-1, IL-6, and interferons (5, 56). Understanding whether CD24 blockade reshapes these cytokine circuits or whether cytokine-driven states condition responsiveness to CD24 targeting will be critical for designing rational combination therapies and predicting therapeutic outcomes (13, 66).

### 6.3 Spatial and temporal profiling to guide precision targeting

Advances in spatial transcriptomics, multiplex proteomics, and single-cell technologies offer unprecedented opportunities to map CD24-mediated immune suppression within the architectural context of tumors (159, 160). Applying these tools longitudinally will help clarify how CD24 expression, Siglec-10/G-positive macrophage distribution, and immune composition evolve during disease progression and treatment (38). Such spatially informed analyses may enable the identification of CD24-dependent immune niches and guide localized or temporally staged therapeutic interventions (84).

### 6.4 Refining biomarker frameworks for clinical translation

Translating CD24-targeted strategies into clinical practice will require robust, standardized biomarker frameworks that are feasible in real-world settings (89, 161). Future efforts should prioritize the development of clinically deployable assays that integrate tumor CD24 expression with myeloid cell phenotyping and functional immune readouts (162). Prospective validation of these biomarkers in early-phase clinical trials will be essential for defining patient populations most likely to benefit from CD24-directed therapies and for avoiding empiric trial designs that obscure therapeutic potential (156).

### 6.5 Designing precision-focused clinical trials

Given the context-dependent nature of CD24-mediated immune suppression, future clinical trials should adopt biomarker-enriched and adaptive designs (163). Such approaches may include stratification based on macrophage abundance or CD24-Siglec pathway activity, incorporation of immune and spatial endpoints, and rational combination strategies tailored to dominant immune resistance mechanisms (84, 139). Importantly, trial endpoints should extend beyond conventional response metrics to capture immunological remodeling and durable disease control (164). Collectively, these future directions emphasize that realizing the full therapeutic potential of CD24 will require coordinated advances in biology, biomarker development, and clinical strategy, paving the way for more precise and effective immunotherapy approaches in solid tumors (21).

**Figure 5:**
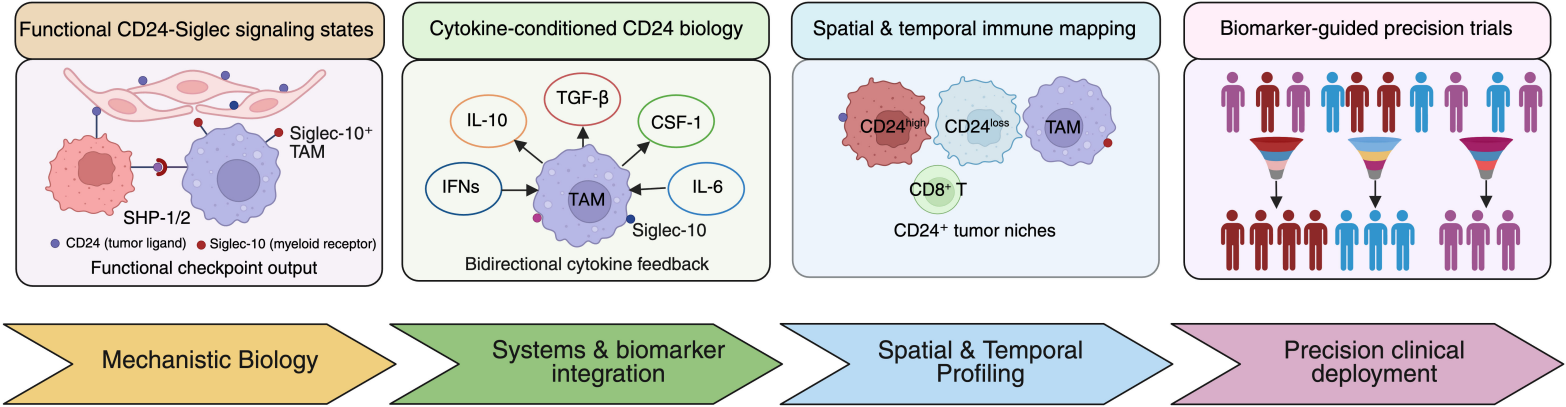
CD24 targeting in next-generation precision immunotherapy. Conceptual framework illustrating future directions for integrating CD24-directed strategies into precision cancer immunotherapy. Functional characterization of CD24-Siglec signaling states, cytokine-conditioned modulation of myeloid biology, and spatial-temporal immune mapping are integrated through systems- and biomarker-level analyses to define CD24-dependent immune niches. These insights inform biomarker-guided patient stratification and precision clinical trial design, enabling rational deployment of CD24-targeted therapies and combination strategies tailored to dominant immune resistance mechanisms.

Accordingly, these converging biological, biomarker, and clinical considerations are summarized schematically in **Figure 5**, which outlines a precision framework for integrating CD24-targeted strategies into next-generation cancer immunotherapy.

## 7 Conclusion

CD24 has emerged as more than a tumor-associated marker, assuming a central role as an innate immune checkpoint that integrates tumor plasticity with myeloid-mediated immune suppression in solid tumors. Advances over the past several years have clarified the mechanistic basis of CD24-Siglec signaling, establishing its capacity to restrain macrophage phagocytosis and to shape the immunological landscape of the TME. These insights have repositioned CD24 as a biologically actionable target with implications that extend beyond tumor-intrinsic behavior to encompass dynamic immune regulation.

At the same time, the expanding therapeutic repertoire targeting CD24 including antibody-based approaches, nanotechnology-enabled platforms, localized delivery systems, and engineered cellular therapies has underscored the context-dependent nature of CD24 biology. The heterogeneity of CD24 expression, redundancy among innate immune checkpoints, and potential for adaptive resistance highlight the limitations of universal or monotherapy strategies. Collectively, these considerations argue against empiric deployment and instead support biomarker-guided approaches that align therapeutic modality with dominant immune suppressive mechanisms within individual tumors.

Looking forward, the successful clinical translation of CD24-directed therapies will depend on integrating functional and spatial biomarkers, cytokine and myeloid signaling dynamics, and rational combination strategies into precision-focused trial designs. By embedding CD24 targeting within a systems-level understanding of the TME, future studies can better define where CD24 represents a critical vulnerability and how its modulation can be leveraged to enhance durable antitumor immunity. Such an approach positions CD24 not as a standalone solution, but as a strategically deployed component of next-generation, personalized cancer immunotherapy.

## Glossary

ADC: Antibody-drug conjugate
CAR-T: Chimeric antigen receptor T cells
CD24: Cluster of differentiation 24
CD44: Cluster of differentiation 44
CD47: Cluster of differentiation 47
CSC: Cancer stem cell
CSF-1: Colony-stimulating factor 1
DAMP: Damage-associated molecular pattern
EMT: Epithelial-mesenchymal transition
FAK: Focal adhesion kinase
GPI: Glycosylphosphatidylinositol
HCC: Hepatocellular carcinoma
ICI: Immune checkpoint inhibitor
IHC: Immunohistochemistry
IL: Interleukin
LILRB: Leukocyte immunoglobulin-like receptor B
mAb: Monoclonal antibody
MDSC: Myeloid-derived suppressor cell
NK: Natural killer
PD-1: Programmed cell death protein 1
PD-L1: Programmed death-ligand 1
PI3K: Phosphoinositide 3-kinase
Siglec: Sialic acid-binding immunoglobulin-like lectin
SHP: Src homology region 2 domain-containing phosphatases
SIRPα: Signal regulatory protein alpha
TAM: Tumor-associated macrophage
TME: Tumor microenvironment.
